# Development and Preliminary Findings of a Modified WHO Caregiver Skills Training Program for Children with Autism in Mainland China

**DOI:** 10.3390/bs16071159

**Published:** 2026-07-09

**Authors:** Rui Meng, Lingyue Kong, WHO CST Team, Chongying Wang

**Affiliations:** 1 Department of Social Psychology, School of Sociology, Nankai University, 38 Tongyan Road, Tianjin 300350, China; 2 Autism Research Center, Nankai University, Tianjin 300350, China; 3 Department of Noncommunicable Diseases and Mental Health, World Health Organization, 1211 Geneva, Switzerland

**Keywords:** autism, parent-mediated intervention, caregivers, World Health Organization Caregiver Skills Training, China

## Abstract

**Purpose:** Most children with autism live in resource-limited settings with limited access to timely interventions. To address this gap, the World Health Organization developed Caregiver Skills Training (CST) to support caregivers and expand intervention access globally. This study examined the feasibility and preliminary outcomes of a modified CST in mainland China. **Methods:** Using the ecological validity model and qualitative interviews, the CST materials were culturally adapted and modified for the Chinese context. A pre- and post-test controlled trial was conducted with caregivers of children with autism aged 2–9 years, who were assigned to either the CST intervention group (*N* = 15) or a caregiver education control group (*N* = 15). Clinical outcomes for caregivers and children were evaluated at baseline and after a 10-week intervention period. **Results:** Cultural adaptation and modifications focused on language adjustments, localization of case examples and demonstrations, and optimization of teaching methods and training schedules. Supplementary within-group analyses indicated pre–post changes in caregiver knowledge and skills, parenting stress, and selected child outcomes, including speech/language/communication, sensory/cognitive awareness, and overall autism symptoms. However, most between-group differences were not statistically significant after baseline adjustment. **Conclusions:** The findings provide preliminary evidence for the feasibility of culturally adapted and modified CST in mainland China. Given the pilot nature of the study and the absence of statistically significant between-group effects for most outcomes, the outcome findings should be interpreted as exploratory and hypothesis-generating rather than evidence of efficacy. Further large-scale studies with greater statistical power and objective outcome measures are needed to evaluate effectiveness and implementation feasibility.

## 1. Introduction

Neurodevelopmental disorders are conditions characterized by impaired brain or central nervous system development, including disabilities such as attention deficit hyperactivity disorder (ADHD), autism spectrum disorder (ASD), and intellectual disability (Jeste, 2015). They constitute significant global public health concerns, primarily due to the considerable economic burden associated with long-term care and support services, including various interventions (Lord et al., 2018; Rogge & Janssen, 2019).

Although early intervention is critical for improving outcomes in children with developmental disorders (Seida et al., 2009), timely diagnosis and access to services remain limited worldwide (Patel et al., 2018). This burden is particularly acute in low- and middle-income countries (LMICs), where approximately 95% of children with developmental disabilities, including ASD, reside according to the (Global Research on Developmental Disabilities Collaborators, 2018). In China, caregiver and public awareness of autism remains limited, and shortages in diagnostic resources and early-intervention services constrain timely identification and support for children with autism (Liao et al., 2022; Sun et al., 2019). Services supporting individuals with ASD are further constrained in China due to inadequate training capacity, a shortage of specialized teachers, rare inclusive services outside major cities, and financial burden on families (Liao et al., 2022; Su et al., 2013; Ye, 2022; Zhou & Xu, 2019).

For these implementation gaps in LMICs, the World Health Organization (WHO), supported by Autism Speaks and collaborators, developed the Caregiver Skills Training (CST) program to strengthen treatment and services for children with NDDs (Salomone et al., 2019). WHO CST is a structured, caregiver-mediated intervention designed for caregivers of children aged 2–9 years with developmental delays or disabilities, including autism. It aims to strengthen caregivers’ ability to support their children’s social communication, engagement, adaptive behaviors, and daily living skills through strategies embedded in everyday routines. The standard WHO CST model includes nine group sessions and three individualized home visits, which can be delivered in community- and home-based settings to improve accessibility in resource-constrained contexts (World Health Organization, 2022a, 2022b). The mechanism of change underlying CST involves both caregiver- and child-level processes. By providing structured training in naturalistic, play-based interaction, using modeling, role-play and constructive feedback, CST enhances caregivers’ skills, confidence, and psychological well-being (World Health Organization, 2026). Improved caregiver competencies subsequently facilitate more responsive and developmentally supportive interactions with their children, which in turn promote children’s adaptive functioning, communication, and social participation (Salomone et al., 2018; Szlamka et al., 2022). In this way, CST operates through a caregiver-mediated pathway, translating intervention content into meaningful changes in daily caregiving practices and family functioning.

The WHO CST has been adaptively implemented and piloted across diverse global contexts, including Italy, South Africa, Ethiopia, Kenya, India, Hong Kong, and Taiwan (Salomone et al., 2022b; Schlebusch et al., 2024; Tekola et al., 2020; Sengupta et al., 2023; Lau et al., 2022; Wong et al., 2022; Seng et al., 2022). Existing evidence suggests that the program demonstrates acceptable feasibility across settings, with emerging evidence of preliminary effectiveness from pre-post studies (Tekola et al., 2020; Sengupta et al., 2023; Lau et al., 2022). A randomized controlled-trial of CST against treatment as usual in the public health system in Italy demonstrated high levels of acceptability, feasibility (Salomone et al., 2022a) and clinical effects on blind rated quality of care-giver-child interaction (Salomone et al., 2022b), with changes in caregiver’s skills significantly mediating the effect of treatment at the 3-month follow-up (Settanni et al., 2024). A large randomized-controlled trial in underway in Ethiopia and Kenya (Washington-Nortey et al., 2024). Importantly, this series of work highlights the critical role of contextual adaptation in maintaining intervention integrity while enhancing cultural relevance.

From a theoretical perspective, cultural adaptation is necessary to ensure that intervention components align with local socio-cultural contexts while preserving core therapeutic mechanisms. Theory-informed frameworks, such as the Ecological Validity Model (EVM; Bernal et al., 1995), provide structured guidance for balancing fidelity and contextual fit. Supporting evidence from systematic reviews further highlights iterative, stakeholder-engaged adaptation processes (Castro et al., 2010; Day et al., 2023). Examples from previous CST studies illustrate these theoretical principles: in Italy, CST was integrated into public child neuropsychiatric services and delivered by trained clinicians within routine care pathways (Salomone et al., 2022a); in low-resource contexts such as South Africa, Ethiopia, Kenya, and India, task-sharing models with non-specialist facilitators were adopted to deliver parent-mediated interventions and culturally relevant caregiver support (Schlebusch et al., 2024; Tekola et al., 2020; Washington-Nortey et al., 2024; Sengupta et al., 2023); and in Hong Kong and Taiwan, adaptations included culturally tailored case examples and hybrid or technology-assisted delivery formats (Lau et al., 2022; Wong et al., 2022; Seng et al., 2022).

From a practical perspective, CST represents a promising approach for addressing service gaps in low- and middle-resource contexts, including mainland China. As an open-access, manualized, and caregiver-mediated intervention, CST does not require a formal diagnosis for participation (Salomone et al., 2019), which may be particularly advantageous in settings where stigma discourages recognition of autism (de Leeuw et al., 2020). Early intervention approaches in China have traditionally emphasized highly structured, adult-directed teaching methods, which are different from CST’s child-led, play-based approach (Liao et al., 2022; McCabe, 2013). Because experiential learning strategies, such as modeling, role-play, practice, and feedback, are core components of WHO CST, the modification process aimed to retain these methods while adjusting language, examples, and delivery arrangements to improve local relevance and feasibility. Once adapted, the standardized and low-cost structure of CST facilitates scalability and may mitigate barriers posed by conventional early intervention services, while group-based delivery further supports caregiver engagement and psychological well-being (Durkin et al., 2015; Huang et al., 2013; Tekola et al., 2020; Salomone et al., 2018). CST is designed for global implementation across diverse cultural, socioeconomic, and resource landscapes following its rigorous contextual adaptation (World Health Organization, 2022a; Salomone et al., 2019). While recent studies have demonstrated its utility in Taiwan and Hong Kong, there remains limited evidence of peer-reviewed research examining the feasibility and preliminary findings of CST within mainland China (Lau et al., 2022; Wong et al., 2022; Seng et al., 2022). Together, these theoretical and practical considerations provide a clear rationale for conducting systematic cultural adaptation in mainland China, highlighting both the necessity of structured frameworks and the practical challenges in local implementation.

To address this gap, the present study focuses on the cultural adaptation and preliminary evaluation of WHO CST in mainland China. In this pilot study, caregiver outcomes were treated as proximal outcomes, reflecting the immediate effect of CST on caregiver skills, confidence, and psychological well-being, which are expected to drive downstream changes in children’s adaptive functioning and social participation (Settanni et al., 2024). Child outcomes were treated as preliminary distal outcomes, reflecting the dependent, exploratory effects of changes in caregiver practices. Theoretically, the study considers the challenges caregivers face when transitioning from traditional, adult-directed, content-driven approaches to child-led, naturalistic play-based interactions. Practically, it examines how culturally adapted support and targeted guidance may facilitate this shift and support caregivers in integrating developmental strategies into daily routines. Specifically, this study had two primary aims: (1) to conduct a systematic cultural and contextual modification of WHO CST for implementation in mainland China, including modifications to intervention materials, delivery approaches, and caregiver engagement strategies, such as translation and language simplification, locally relevant examples, adjusted session activities, flexible delivery arrangements, and support for caregiver participation (Phase 1), and (2) to evaluate its feasibility, and preliminary effects of the adapted and modified intervention on child-related outcomes (e.g., autism symptoms were treated as the primary outcome, while speech/language/communication, sociability, sensory/cognitive awareness, and health/physiology/behavior were analyzed as secondary outcomes) and caregiver-related outcomes (e.g., mental health, knowledge and skills, self-efficacy, and parenting pressure) (Phase 2). Feasibility was assessed through indicators of recruitment, retention, session attendance, and implementation safety. By addressing both adaptation and outcome-related questions, this study aims to provide initial empirical evidence to inform the broader dissemination of caregiver-mediated interventions in mainland China.

## 2. Phase 1: Cultural Adaptation and Modification of WHO CST in Mainland China

### 2.1. Method

#### 2.1.1. Study Design and Cultural Adaptation and Contextual Modification Framework

From December 2023 to March 2024, the Chinese cultural adaptation and modification of the CST were conducted using a multi-phase qualitative design with a primary focus on linguistic equivalence and ecological validity. This process involved a formal translation/back-translation protocol followed by iterative stakeholder engagement, including one focus group discussion, two stakeholder consultation meetings, and seven in-depth interviews, as recommended by WHO (World Health Organization, 2022a). The CST manuals (World Health Organization, 2022a, 2022b, 2022c, 2022e) were translated into Chinese and subsequently back-translated into English by postgraduate students specializing in developmental disorders, under the supervision of doctoral-level advisors. Throughout this process, the translation teams systematically examined linguistic nuances, contextual considerations, and cultural distinctions to ensure semantic and contextual accuracy. Subsequently, the study applied the Ecological Validity Model (EVM; Bernal et al., 1995) as a guiding framework for the cultural and contextual modification of WHO CST. In this study, cultural adaptation was understood as a planned form of intervention modification intended to improve cultural and contextual relevance, whereas intervention modification was used as a broader term referring to changes in materials, delivery approaches, session structure, duration, caregiver engagement strategies, and implementation supports. These definitions informed the development of inquiry protocols for one focus group discussion, two stakeholder consultation meetings, and seven one-on-one in-depth interviews, which explored potential modifications to intervention materials, delivery approaches, and caregiver engagement strategies in the mainland Chinese context.

#### 2.1.2. Participants

A purposive maximum variation sampling strategy was employed to recruit information-rich participants across three stakeholder domains: Lived Experience, Frontline Implementation, and Systemic Oversight. Participants in this study included caregivers of children with autism (*n* = 9), directors of rehabilitation institutions (*n* = 2), therapists (*n* = 6), postgraduate students specializing in psychology (*n* = 2), and a child psychiatrist (*n* = 1) (as shown in Table 1).

Caregivers with lived experience were required to be primary caregivers of at least one child with autism aged 2–9 years, reflecting the critical post-diagnostic intervention period and enabling assessment of the intervention’s cultural relevance and ecological fit within family contexts. Frontline implementation perspectives were provided by therapists and postgraduate psychology students, each with a minimum of two years’ experience delivering autism-related rehabilitative interventions. This group assessed the technical feasibility and clinical appropriateness of the translated materials. System-level oversight was represented by two rehabilitation directors with at least five years of managerial experience in autism services and one child psychiatrist with over five years of clinical experience working with children aged 2–9 years and their families. These stakeholders contributed expertise regarding local caregiver needs, policy and resource constraints (e.g., staffing, venue, and training capacity), clinical governance, and the integration of post-diagnostic support pathways. This multi-stakeholder triangulation ensured that the cultural adaptation process was grounded in clinical standards, implementation feasibility, and local sociocultural realities.

#### 2.1.3. WHO CST Procedures and Materials

CST is delivered using a cascade training model in which certified Master Trainers may directly support caregivers or train non-specialist facilitators, who subsequently deliver the intervention to caregivers. This model facilitates broader delivery of caregiver-mediated intervention strategies while supporting implementation fidelity and scalability. This structure ensures fidelity while remaining flexible enough for trainers to provide direct guidance to caregivers when necessary.

Each session is structured to integrate instruction, demonstration, and guided practice. Caregivers observe modeling of strategies, engage in role-plays, and discuss challenges and experiences applying the strategies in their daily routines. Home visits provide individualized support, reinforce newly acquired skills, and help caregivers establish practical goals for implementing strategies at home. Regular home practice between sessions reinforces skill acquisition, facilitates generalization to daily life, and allows iterative feedback and problem-solving. The overall design emphasizes not only caregiver skill development but also the support of caregiver well-being and problem-solving abilities (Salomone et al., 2019).

CST is designed to be broadly applicable across the 2- to 9-year-old age range by setting different intervention goals and strategies according to each child’s developmental level. Play activities, for example, are organized into progressive levels—simple play, put-together play, early pretend play, and advanced pretend play—allowing caregivers to practice strategies appropriate to their child’s abilities. Materials and activities are selected to match these levels; for instance, a put-together play activity might involve throwing balls into a bucket, whereas an advanced pretend play activity could involve pretending the ball is a “hot cake” taken out of the oven. By adjusting both the goals and the strategies to the child’s developmental stage, CST facilitates meaningful engagement, supports skill acquisition, and ensures that the intervention is relevant and effective for children across the full age range (Salomone et al., 2019; Tekola et al., 2020).

During Phase 1, the CST materials were culturally adapted and revised to ensure that they were understandable, contextually appropriate, and meaningful for the local caregiver population. Guided by stakeholder feedback and the Ecological Validity Model (EVM) framework, these adaptations focused on refining language, examples, and contextual elements while maintaining the integrity of the program’s core strategies and principles. These revisions provide a foundation for later phases of implementation and evaluation while preserving the original pedagogical structure and intended outcomes of the CST.

#### 2.1.4. Interview Procedure and Data Collection

Upon obtaining informed consent, data were collected through focus groups, stakeholder consultation meetings, and individual in-depth interviews. These were conducted either online or face-to-face in accordance with participants’ preferences, and all sessions were audio-recorded to ensure accuracy. A semi-structured interview guide was developed to systematically explore participants’ perspectives on the WHO CST materials, including domains such as content, language, and course structure, as well as procedural aspects of implementation (e.g., session frequency, training setting, supplementary services, supervisory mechanisms, and delivery models).

For the qualitative interviews, a purposive sample was used to capture diverse perspectives from caregivers of children with autism, therapists, and psychology postgraduate students familiar with the WHO CST. A single focus group (*n* = 7) was conducted online via Tencent Meeting to elicit feedback on cultural adaptation. Participants reviewed WHO CST materials in advance and engaged in a structured discussion lasting 100–120 min. Two stakeholder consultation meetings were conducted at different autism rehabilitation institutions. The first meeting included four participants and the second included two, comprising directors of rehabilitation institutions, child psychiatrists, and therapists. Each session lasted approximately 80–100 min and was audio-recorded using the “Xunfei Hearing” App v6.0 (Voice-to-text transcription software). These sessions aimed to identify systemic needs and challenges in clinical practice and to gather suggestions for adapting CST materials and delivery.

Furthermore, seven caregivers were recruited for individual in-depth interviews using a maximum variation sampling strategy based on age, gender, educational attainment, and the developmental stage of their children. A semi-structured guide was employed, with topics tailored to each interviewee, covering areas such as children’s intervention experiences, caregivers’ roles, prior training participation, perceived benefits and challenges, needs and barriers to training, and recommendations for adapting the WHO CST. Example questions explored which components of WHO CST were most relevant or engaging, whether the content and delivery format met caregivers’ needs, and which aspects of the intervention could be modified to improve cultural relevance, contextual fit, and implementation feasibility. Each interview lasted 30–40 min and was audio-recorded using the “Xunfei Hearing” App. Researchers organized the focus group discussions, stakeholder meetings, and individual interviews according to the type of participants and the format of each session, and the interview questions were flexibly adjusted as needed to suit participants’ responses and ensure comprehensive coverage of relevant domains.

#### 2.1.5. Data Analysis

All sessions were audio-recorded with participants’ consent, transcribed verbatim, and verified within 24 h. Data collection continued until thematic saturation was reached. Qualitative data were systematically organized and analyzed using NVivo 15.0, guided by the six-step thematic analysis framework proposed by Braun and Clarke (2006), and grounded in the theoretical foundation of the EVM. Coding was iterative: initial engagement with the transcripts prioritized semantic-level coding to generate preliminary nodes. During theme development, codes were classified and collated, and potential subthemes and overarching themes were identified with reference to the ecological validity model. Coded extracts under each candidate theme were then re-read to ensure coherent fit with the original data and to consolidate themes. Theme conceptualization was grounded in existing theoretical frameworks and literature, with labels refined to be both descriptive and comprehensive. Selected FRAME elements (Wiltsey Stirman et al., 2019) were used after theme development to describe the primary modification focus of each theme, including what was modified, the rationale for modification, and potential fidelity considerations. FRAME was used as a reporting aid rather than as a deductive coding framework. In the reporting phase, relationships among themes were explicated by integrating analytic narrative with illustrative excerpts, attending to inter-theme influences, and deriving strategies for cultural adaptation and revision.

Following the qualitative analysis, all identified themes were systematically linked to potential adaptations in the CST. Feedback from caregivers and stakeholder meetings was reviewed collectively by the research team, and iterative discussions were conducted to determine how each theme could inform modifications to language, illustrative examples, metaphors, delivery approaches, and caregiver engagement strategies. All decisions were documented to maintain transparency and a clear rationale for the adaptations. The adaptation and modification process was conducted using a structured, iterative review approach guided by EVM. Although formal inter-coder reliability and multi-source triangulation were not conducted, regular team discussions and a consensus-based review process supported the rigor and credibility of the qualitative analysis.

To maintain continuity between the two study phases, findings from Phase 1 were used to refine the CST materials and delivery procedures prior to pilot implementation. The resulting adaptations, such as modifications to language use, culturally relevant examples, delivery formats, and caregiver engagement strategies, enable the Phase 2 pilot to assess feasibility and preliminary outcomes while maintaining the fidelity to the core principles of the original CST.

### 2.2. Results

#### 2.2.1. Cultural Adaptation and Intervention Modification

Guided by the Ecological Validity Model (EVM), the qualitative analysis identified eight major themes, 48 subthemes, 110 primary codes, and 369 meaning units (see Table 2). These findings informed a systematic process of cultural adaptation and intervention modification for the Caregiver Skills Training (CST) program developed by the World Health Organization. The adaptations addressed multiple EVM dimensions, including language, metaphor, content, context, method, person, and goal—and involved both surface-level adjustments (e.g., terminology, examples, and visual representations) and deeper structural modifications (e.g., adult learning strategies, program structure, and caregiver engagement mechanisms). Throughout the adaptation process, careful attention was paid to maintaining fidelity to the core principles and therapeutic processes of the original CST model, ensuring that adaptations focused on improving cultural relevance, accessibility, and feasibility within the local socio-cultural context. The key adaptation themes and corresponding program modifications are presented in the following sections.

In addition to the EVM-based organization of themes, the primary modification focus of each theme was summarized with reference to selected FRAME elements. Using selected FRAME elements to describe the primary modification focus, most language-, concept-, and metaphor-related themes reflected material- or terminology-level modifications intended to improve comprehensibility and cultural acceptability. Persons-, context-, and goals-related themes mainly reflected caregiver engagement, barrier reduction, and individualized goal-setting supports. Methods-related themes involved delivery-process modifications, such as changes to recruitment, course structure, time allocation, delivery format, and follow-up support, which may require further evaluation to ensure fidelity to the core components of WHO CST.

Some identified modifications were consistent with adaptations recommended in the WHO Adaptation and Implementation Guide, such as localizing character names, terminology, examples, illustrations, and delivery arrangements to improve comprehensibility, acceptability, and feasibility. Other themes reflected locally identified modifications that extended beyond the recommended adaptations, including attention to intergenerational caregiving roles, family privacy during home visits, alignment of family goals, caregiver motivation, online community support, and supplementary caregiver psychological support needs.

#### 2.2.2. Linguistic Refinement and Symbolic Adaptation (Language, Metaphor)

Adaptations within the Language and Metaphor dimensions aimed to reduce cultural distance and enhance the clarity and relatability of intervention materials. Common local names were adopted throughout the materials (G1-P4, G1-P1, G1-P6) as per WHO guidance (World Health Organization, 2022a). Intergenerational terminology was also adjusted to reflect local affectionate norms; for example, formal references to “grandparents” or “father and mother” were replaced with colloquial, culturally familiar iterations such as “mom and dad, grandma, grandpa, maternal grandma, and maternal grandpa” (G1-P1).

Beyond nomenclature, professional stakeholders emphasized the need for “concise and focused” textual content. To mitigate comprehension barriers posed by “obscure or overly technical terminology,” the adapted materials employ simplified written vocabulary, supplemented by oral explanations designed to be easily understood by caregivers with varying educational backgrounds (G3-T1).

In the Metaphor dimension, symbolic characters were localized to align with the regional socio-cultural landscape. This modification was consistent with WHO-recommended material-level adaptations, which encourage replacing culturally specific roles and examples when they are not locally familiar. Stakeholders noted that “the role of a priest is actually quite rare” in the local context (G1-P1); therefore, the priest character was replaced with a doctor (G2-T1), a professional role more familiar to caregivers in the local healthcare context. These modifications ensured that illustrations, story characters, and scenarios reflected local cultural norms, enhancing participant engagement, comprehension, and relatability.

#### 2.2.3. Localization of Narrative and Situational Context (Content)

Modifications within the Content dimension primarily aimed to improve the practical applicability and developmental appropriateness of the intervention. Stakeholders indicated that several components of the original CST framework were viewed as “redundant and repetitive,” particularly within the child engagement modules (G1-P6). In response to caregivers’ substantial time constraints, the original nine-session curriculum was condensed into five integrated units. This restructuring retained the fundamental principles and theoretical foundations of the CST model while reorganizing the learning sequence to enhance efficiency and coherence (G1-P1). The detailed reorganization is presented in Table 3.

In addition to structural modifications, the revised curriculum incorporated enhanced developmental guidance. Caregivers reported difficulty in “assessing the effectiveness of specific strategies” due to a lack of reference points for assessing their child’s developmental level (G1-P3). To address this issue, the adapted program integrated structured information on developmental milestones, enabling caregivers to set realistic expectations and goals that correspond to their child’s developmental stage (G1-P5).

Furthermore, deep-structure cultural adaptations were applied to the program’s narrative and visual components. Participants described certain Western-based stories and images as “distant and difficult to relate to.” Consequently, these materials were revised to incorporate culturally relevant caregiving scenarios, familiar family interaction patterns, and references to local service systems and public welfare resources (G1-P1, G1-P6), while the original illustrations were retained.

Within the Context dimension, socio-economic and logistical barriers emerged as critical factors influencing participation. Directors noted that many families rely on a single primary caregiver who is unable to attend training due to a lack of childcare support (G3-T1). To mitigate these structural constraints, the modified WHO CST incorporated on-site childcare services during training sessions. Additionally, a WeChat-based communication group was used as a supplementary channel to support ongoing caregiver communication and provide flexible contact for caregivers facing transportation, employment, or childcare-related barriers (G1-P6). This represented a context-specific implementation support using an existing social media tool, rather than a newly developed digital platform. These contextual modifications were designed to enhance accessibility, reduce participation inequities, and support sustained engagement in the intervention. As a result, the modified CST curriculum was reorganized into five integrated training sessions, with examples and developmental guidance revised to improve clarity, cultural relevance, and practical usability for caregivers.

#### 2.2.4. Pedagogical Optimization and Goal Realignment (Method, Goal)

Findings within the Method dimension highlighted the importance of preserving WHO CST’s experiential adult learning approach when adapting the delivery format and integration session content. Stakeholders emphasized that, although WHO CST includes guided discussion, modeling, caregiver practice, feedback, coaching, home practice, and subsequent review, these components needed to be maintained in the modified delivery format to prevent caregivers from developing only a “superficial understanding” of intervention strategies (G1-P2). In response, the modified version places greater emphasis on real-time modeling, guided rehearsal, and interactive participation. For example, therapists recommended adding an explanatory distinction between “genuine praise” and praise expressed mainly through heightened emotion, which was not specified in the published version of WHO CST. This distinction was demonstrated through exaggerated facial expressions, tone modulation, and embodied cues to make the praise strategy more intuitive and easier for caregivers to apply in everyday interactions (G2-T2, G2-D1).

Within the Goal dimension, adaptation extended beyond skill delivery to the recalibration of intervention priorities. While the original WHO CST emphasizes general behavioral management and caregiver-mediated interaction strategies, local caregivers identified “school adaptation” as a pressing concern, particularly for children nearing school entry (G1-P6). Accordingly, the curriculum was modified to incorporate individualized ability assessments and structured goal-setting procedures to align intervention objectives with educational readiness (G1-P7).

In addition, professional stakeholders underscored the psychological burden faced by families, including “stress, scarcity of resources, and stigmatization” (G2-T1, G3-P1). To address these contextual pressures, group-based psychological support components were incorporated, supplementing the original self-care guidance with structured peer counseling and facilitated discussion. Consequently, the adapted program expanded experiential learning components, including facilitator-guided rehearsal, role-play, and personalized goal setting, to strengthen caregivers’ practical skill acquisition and better address locally identified priorities such as school readiness and caregiver well-being.

#### 2.2.5. Structural Reorganization and Training Framework (Method, Context)

Adaptations within the Method and Context dimensions resulted in a comprehensive restructuring of program delivery. Building on the flexibility already allowed within WHO CST delivery, the stakeholder-driven modifications focused on improving local feasibility, accessibility, and sustained engagement through adjustments to recruitment strategies, scheduling arrangements, caregiver support, and delivery-related implementation supports.

##### Targeted Recruitment and Stratification

To increase cohort homogeneity, stakeholders proposed narrowing the participant eligibility range from the original 2–9 years to 2–6 years. Stakeholders further recommended stratifying children into narrower developmental groups (e.g., 2–3 years and 3–6 years) and strengthening collaboration with developmental pediatrics departments and disability support organizations. Such strategies were proposed to ensure that training content corresponds more closely with stage-specific developmental needs (G1-P6, G3-T1). Nevertheless, to maintain the intervention’s reach and maximize the benefit for caregivers during this pilot phase, no further exclusionary restrictions were implemented.

##### The Hybrid Intensive Model

To address conflicts between caregiving responsibilities and weekly attendance requirements, a hybrid intensive delivery model was proposed. Compared with the original CST structure, the adapted program consolidated the nine-session curriculum into five integrated modules, reorganizing the learning sequence to improve efficiency and coherence. Delivering the five face-to-face integrated modules within a condensed timeframe was also considered beneficial for maintaining motivation and reducing attrition among families (G1-P5, G3-T1).

##### Hybrid Support and Supervision

Stakeholders also identified several practical barriers affecting program participation. Many caregivers reported that childcare responsibilities limited their ability to attend in-person sessions, particularly for families in which a single caregiver was responsible for the child (G3-T1). Transportation constraints and work schedules were also noted as barriers to regular attendance. At the same time, participants emphasized the importance of maintaining opportunities for direct interaction with facilitators and peer discussion during the training process (G1-P2). These findings highlighted the need for a more flexible delivery model that could improve accessibility while preserving opportunities for personalized guidance and experiential learning.

In response, the adapted program adopted a hybrid delivery structure combining in-person and remote components. On-site childcare support was incorporated to facilitate caregiver attendance during 5-week group training sessions. The modification did not involve creating a new structured sequence of home visits, as WHO CST already recommends individualized visits before the first group session, during the middle of the group sessions, and after the final group session. Instead, the main delivery modification was the addition of a five-week intensive practice phase, consisting of twice-weekly 50 min individualized coaching sessions. During this phase, there were twice-weekly 50 min individualized sessions with real-time feedback as follow-up. In addition, online participation options were introduced during the follow-up intensive practice phase via videoconferencing platforms to accommodate caregivers who were unable to attend in person. These options provide “*greater flexibility in scheduling*” and eliminate travel-related barriers (G1-P1). To maintain opportunities for individualized interaction during the 5 integrated modules, in-person group sessions were limited to fewer than ten participants enable “*in-depth discussion of individual needs*” (G1-P2).

##### Long-Term Reinforcement Mechanisms

Stakeholders further emphasized the importance of sustaining intervention strategies after the formal training period ended. Caregivers expressed concerns that without continued guidance it might be difficult to consistently apply newly learned techniques in daily caregiving routines (G3-P5). Professionals also highlighted the need for ongoing learning opportunities as children’s developmental needs evolve (G3-T1). These perspectives underscored the importance of embedding mechanisms that support continued practice and reinforce skill use beyond the structured intervention period.

Stakeholders emphasized the need for follow-up training sessions (P2) to provide ongoing supervision, peer exchange, and troubleshooting after the core training sessions. Such sessions were suggested as a way to review caregivers’ use of CST strategies at home, address implementation difficulties, reinforce key principles, and provide additional guidance as children’s developmental needs changed. Caregivers also received the WHO CST participants’ guide to support the use of intervention strategies within home routines. These insights suggested that future implementation may benefit from supplementary communication support and follow-up guidance to enhance accessibility and sustain caregiver engagement.

#### 2.2.6. Family Engagement and Caregiver Participation (Person)

Findings within the Person dimension highlighted the importance of recognizing the diversity of caregiving roles within families and encouraging the participation of multiple family caregivers in the training process. Stakeholders emphasized that caregiving responsibilities are often shared among several family members, highlighting the importance of implementing the WHO CST recommendation to invite two caregivers per family whenever possible (World Health Organization, 2022d). This was considered particularly relevant in the mainland Chinese context, where grandparents, fathers, and other family members may play important roles in daily caregiving and the home-based application of CST strategies.

In particular, rehabilitation center directors noted that fathers tend to participate less frequently in caregiver training programs, despite their important role in child development. As one director explained, “*When recruiting, families are welcome to participate together*” and additional efforts should be made to encourage paternal involvement in the CST (G3-T1). Another stakeholder observed that “*moms are usually more tuned in to learning these skills, while dads may be less involved,*” highlighting the need to actively invite fathers to participate in the training (G3-T1).

Professionals also emphasized the culturally significant role of grandparents in childrearing within the local context. In many families, grandparents provide daily childcare while parents are working, making them key figures in the child’s developmental environment. As one participant suggested, “*We should also include grandpa, grandma, mom’s parents, and dad’s parents as part of the training program for caregivers*” (G1-P1). Another stakeholder noted that it is common for children to be cared for by both parents and extended family members, particularly older relatives, during parents’ working hours (G3-T1). In response to these findings, the adapted program encourages the participation of multiple family caregivers in CST sessions whenever possible.

In addition to broadening caregiver participation, stakeholders highlighted the importance of building supportive relationships between facilitators and caregivers. Directors and interventionists emphasized that establishing emotional connection and trust can enhance caregiver engagement and learning. For example, one facilitator suggested allocating time at the beginning of the program for caregivers to become familiar with the trainers and build rapport (G3-T2). Another participant noted that trainers should actively cultivate personal connections with both parents and older caregivers attending the sessions (G3-T1). These relational strategies were incorporated into the adapted program to foster a supportive learning environment and strengthen caregiver engagement throughout the training process.

Collectively, the modifications detailed across the four thematic areas represent a novel intervention largely inspired from the contents and methods of the CST. By addressing linguistic nuances, domestic caregiving realities, and structural barriers to participation, the adapted Chinese CST (C-CST) ensures a higher degree of ecological validity than the original framework. These results demonstrate that the transition from ‘surface structure’ translation to ‘deep structure’ adaptation is essential for maintaining clinical efficacy while ensuring the feasibility of implementation within the Chinese healthcare ecosystem. Accordingly, the adapted and modified program encourages the participation of multiple family caregivers, such as fathers and grandparents, and incorporates relationship-building strategies between facilitators and caregivers to strengthen family-level implementation of CST strategies.

## 3. Phase 2: Pilot Study

### 3.1. Method

#### 3.1.1. Study Design and Settings

Building on the insights and adaptations from Phase 1, Phase 2 examined the feasibility and preliminary effectiveness of culturally adapted and modified CST within the Chinese context. The pilot implementation of CST in mainland China, conducted from June 2024 to February 2025, employed a controlled pre–post test design to evaluate its initial efficacy. The pilot trial ensured that both the intervention content and delivery procedures were tailored to local needs while preserving the integrity of the original CST. All data collection procedures were reviewed and approved by the Medical Ethics Committee of Nankai University (NKUIRB2023159).

#### 3.1.2. Participants

A convenience sample of 45 child-caregiver dyads, including children diagnosed with autism, was recruited in March 2024, in accordance with the recommended sample size guideline for pilot studies (≥30) (Lancaster et al., 2004). Inclusion criteria for children with autism were: (1) a clinical diagnosis of ASD provided by qualified clinicians (child psychologists, psychiatrists, or pediatricians); (2) age between 2 and 9 years; and (3) autism severity classified as mild or moderate, based on clinical diagnosis, special education teacher observations, and Autism Treatment Evaluation Checklist (ATEC) scores: 20–49 indicate mild and 50–79 indicate moderate autism. Caregivers were eligible if they: (1) were currently raising one or more children with autism aged 2 to 9 years; (2) possessed proficiency in Mandarin, including basic literacy skills, willingness to complete study questionnaires, and ability to use WeChat for community learning and discussions; and (3) voluntarily consented to participate, with informed consent obtained from both caregivers and children. Participants were excluded if they met any of the following conditions: (1) provided inaccurate or incomplete contact information, or expressed unwillingness to participate in the CST; or (2) were concurrently enrolled in other clinical trials involving psychological interventions or skills training for parents or caregivers.

Interested parents were invited to complete an online eligibility screening via QR code. Eligible participants were subsequently contacted by researchers for in-person meetings, during which the study’s objectives, procedures, participants’ rights, benefits, and risks were explained, and any questions were addressed. Informed consent was obtained from all participants following comprehensive disclosure of the program details. After eligibility screening and informed consent, participants were randomly allocated to either the CST group or the control group using a computer-generated randomization sequence. The sequence was generated and managed by an independent researcher who was not involved in intervention delivery. Group assignments were not disclosed to the research team until the point of allocation. To minimize allocation bias, intervention facilitators were not involved in generating the randomization sequence or determining participant allocation. Due to the nature of the behavioral intervention, blinding of participants and facilitators was not feasible.

#### 3.1.3. Feasibility and Sample Size Justification

**Figure 1:**
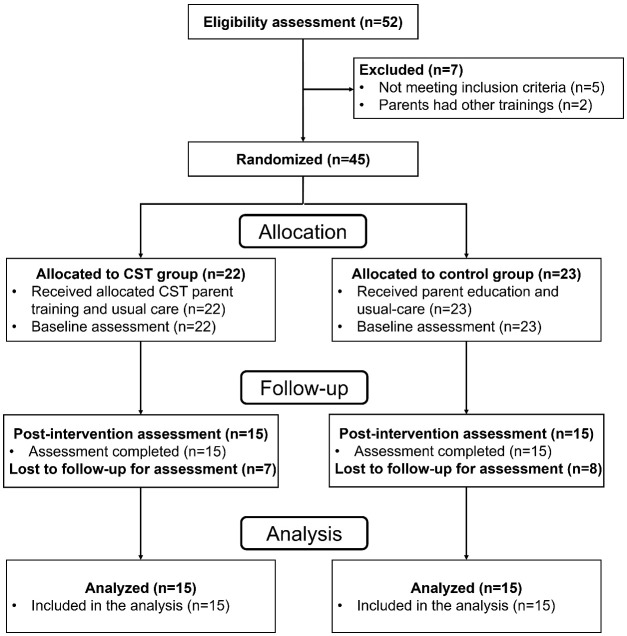
The flow chart of the pre- and post-intervention controlled trial.

The feasibility of the CST was evaluated across several domains, including recruitment, acceptability, usability, retention, adherence, and implementation safety. Recruitment was assessed based on the proportion of eligible participants who provided informed consent and enrolled in the study. Retention and usability were evaluated according to participants’ completion of intervention sessions and pre- and post-intervention assessments. Attrition reasons and adverse events were also documented throughout the intervention period to assess implementation feasibility and acceptability. Participants who completed baseline assessments but did not provide post-intervention data were excluded from the final analysis. Missing post-intervention data were handled using a complete-case (per-protocol) approach (see Figure 1).

Despite attrition, the final sample size of 15 participants per group exceeded the commonly recommended minimum of 12 participants per arm for pilot studies (Julious, 2005), supporting the feasibility-oriented objectives of the present trial. Given the pilot nature of the study and the final analyzed sample size (*N* = 30), the study was primarily intended to evaluate feasibility and preliminary intervention trends rather than to provide definitive tests of between-group efficacy. A post hoc sensitivity analysis indicated limited power to detect medium-sized between-group effects.

#### 3.1.4. Baseline Characteristics

Caregivers in the CST group had a mean age of 35 years (range: 31~41, *N* = 15) and those in the control group 36 years (range: 32~42, *N* = 15). Male participants accounted for one in the CST group and two in the control group. Mothers comprised the majority of caregivers (CST: 80%; control: 66.7%), followed by grandparents (13.3% in both groups). Most participants had an education level of high school or below/diploma (66.7% in both groups). Over 90% reported a monthly household income <15,000 Yuan, with the majority earning 5000–15,000 Yuan (>66.7% in both groups) (as shown in Table 4).

Children with autism in the CST group had a mean age of 6.15 years (SD = 1.59), while those in the control group had a mean age of 6.08 years (SD = 1.29). The male-to-female ratio was 2:1 in both groups. Most children attended special education institutions (CST: 60%; control: 66.7%), followed by kindergartens (CST: 33.3%; control: 26.7%). The mean weekly intervention duration was 14.53 h (SD = 6.99) in the CST group and 16.47 h (SD = 8.17) in the control group.

#### 3.1.5. Data Collection Procedure

Data were collected at two time points: baseline (T0) and immediately post-intervention (T1), within one week of completion. At T0, participants completed online surveys with assistance from researchers, which included demographic information, assessments of autism severity in children with autism, and caregiver-related outcomes (mastery of WHO caregiver knowledge and skills, mental health status, self-efficacy, and parenting stress). At T1, outcome measures for both children with autism and their caregivers were obtained for all participants in both groups.

#### 3.1.6. Intervention Group

In addition to regular institution-based training, children in the CST group received home-based intervention delivered by their caregivers. The intervention group participated in a 10-week culturally adapted and modified CST consisting of two phases from April 2024 to June 2024: (1) five weekly 3 h face-to-face group sessions, and (2) five weeks of twice-weekly 50 min home visits or online videoconferencing via Tencent Meeting. Group sessions consolidated thematically related modules into five core topics (Table 3) and incorporated homework review, Q&A, story discussions, skill demonstrations, role-play, practical exercises, and targeted assignments. The home-based phase provided individualized guidance, assessed children’s play, language, and behavioral skills, and evaluated caregiver proficiency through direct observation. Caregivers were assisted in setting intervention goals and applying CST techniques to implement effective home-based interventions.

##### Facilitators and Fidelity Monitoring

The intervention was implemented by the first author, who holds a Master’s degree in psychology and has extensive experience in training and delivering interventions for children with autism, and served as the primary facilitator for all group sessions and home visits. Two postgraduate psychology students assisted as non-specialist facilitators. All three facilitators were supervised by two experienced Master Trainers throughout the pilot phase. To support implementation consistency, facilitators received standardized session materials and followed structured operational procedures across sessions. Intervention fidelity was assessed using a self-designed fidelity checklist and scoring system developed based on the core components of the CST. The checklist assessed whether the key messages of each CST session were delivered as planned, including session content, key CST strategies, caregiver practice activities, facilitator feedback, and completion of session objectives. Fidelity scores were calculated as the percentage of completed components among all applicable components for each session. A subset of sessions was independently rated by two research team members familiar with the CST and fidelity checklist, and inter-rater agreement reached 85%.

#### 3.1.7. Control Group

Children in the control group received only the regular curriculum-based training provided at their schools or institutions and were not required to participate in home-based caregiver-led interventions. Institutional services for children with autism were based on applied behavioral analysis and encompassed teacher–caregiver communication concerning the children’s homework practice and daily performance. These services also included routine interventions targeting language development, problem behaviors, and perceptual skills, which were consistent with the regular institution-based interventions provided in the CST group. Additionally, caregivers in the control group attended a 10-week educational program consisting of ten sessions online (180 min each), delivered in a lecture format. The sessions provided foundational information on autism characteristics, diagnostic criteria, early intervention strategies, and evidence-based practices, and offered caregivers opportunities to discuss their personal experiences. No skill practice, role-play, or individualized coaching was included.

#### 3.1.8. Measurements

The CST is a caregiver-mediated intervention, and its effectiveness requires evaluation based on outcomes from both caregivers and their children with autism to comprehensively assess effectiveness. For caregivers, the study examined changes in knowledge and skills, self-efficacy, and parenting stress following the training. For children with autism, the study assessed whether parental application of the acquired skills resulted in changes in autism-related symptoms.

With respect to CST practice and home visits, the online home practice guidance offers step-by-step instructions on the essential knowledge and skill components covered in group training, presented in text, video, and audio formats. Home visits or one-on-one video consultations are conducted to evaluate caregivers’ implementation of these skills during interventions with their children. Evaluations are carried out using the home visit scoring sheet from the CST toolkit, accompanied by detailed practical demonstrations and tailored guidance based on specific scoring criteria.

##### Autism Treatment Evaluation Checklist (ATEC)

Autism symptoms were assessed using the Autism Treatment Evaluation Checklist (ATEC; Fang et al., 2019), a 77-item instrument comprising four subscales: speech/language/communication, sociability, sensory/cognitive awareness, and health/physiology/behavior. Scoring formats vary by subscale, but both the total scores and subscale scores indicate that higher scores represent greater symptom severity. The scale demonstrated high internal consistency (Cronbach’s α = 0.91).

##### Mental Health

Caregivers’ mental health was evaluated using the 12-item General Health Questionnaire (GHQ-12; Zhang et al., 2008). The instrument comprises statements addressing various aspects of daily living, requiring respondents to reflect on their experiences over the preceding month. Each item is rated on a 4-point Likert scale ranging from 0 (never) to 3 (almost always), with six items reverse-scored. Total scores range from 0 to 36, with higher scores indicating greater mental health difficulties. This scale demonstrated high internal consistency, as indicated by Cronbach’s α coefficient of 0.88.

##### Caregiver Knowledge and Skills

The degree to which caregivers mastered knowledge and skills was assessed using the WHO Caregiver Knowledge and Skills Test, originally developed by the World Health Organization Caregiver Skills Training Project Research Group and adapted into a Chinese version (unpublished, Chen et al., 2023). The scale consists of 24 items divided into four subscales: dealing with children’s challenging behaviors, the importance of praise, children’s learning opportunities, and self-care. Each item is rated on a 5-point Likert scale ranging from 1 (strongly disagree) to 5 (strongly agree). Total scores range from 24 to 120, with higher scores indicating greater mastery of the knowledge and skills by caregivers (Seng et al., 2022). This scale demonstrated high internal consistency, as indicated by a Cronbach’s α coefficient of 0.83.

##### Caregiver Self-Efficacy

Caregiver self-efficacy was measured using the Caregiver Self-efficacy Questionnaire (CSQ), developed by the World Health Organization Caregiver Skills Training Project Research Group and adapted into Chinese (unpublished). The CSQ consists of 13 items assessing caregivers’ confidence in applying the skills and strategies taught in CST. Items are rated on a 5-point Likert scale (1 = “not confident at all” to 5 = “strongly confident”), yielding a total score of 13–65, with higher scores indicating greater confidence in applying the relevant knowledge and skills. The scale demonstrated good internal consistency in this study (Cronbach’s α = 0.92).

##### Parental Stress

Parental stress was measured using the Parenting Stress Index–Short Form (PSI-SF), a 36-item questionnaire consisting of three subscales: parental distress, dysfunctional parent–child interaction, and difficult child characteristics (Abidin, 1990; Qin et al., 2008). Each item is rated on a 5-point Likert scale, yielding a total score ranging from 36 to 180, with scores exceeding 90 indicating elevated levels of parental stress. The scale exhibited good internal consistency in the present study (Cronbach’s α = 0.85).

#### 3.1.9. Statistical Analysis

Data analysis was conducted using SPSS 26.0. Visual inspection of Q–Q plots indicated that the distributions of the outcome variables did not show substantial deviations from normality. Homogeneity of variance was assessed using Levene’s test. Baseline characteristics between the CST and control groups were examined using chi-square tests for categorical variables (e.g., sex of children and caregivers) and independent-samples *t*-tests for continuous variables (e.g., age and baseline outcome measures of children and caregivers). The primary analyses of intervention effects were conducted using analysis of covariance (ANCOVA), with post-intervention scores as the dependent variables, group (CST vs. control) as the fixed factor, and corresponding baseline scores as covariates. This approach allowed for adjustment of initial differences in symptom severity and developmental functioning. The autism symptom was treated as the primary outcome, while subscale scores (speech/language/communication, sociability, sensory/cognitive awareness, and health/physiology/behavior) were analyzed as secondary outcomes. Within-group changes from pre- to post-intervention were examined using paired-samples *t*-tests as supplementary analyses to describe the direction and magnitude of change. However, the primary analysis regarding intervention effects was based on the ANCOVA results. The same analytic procedures were applied to caregiver outcomes. In addition, the homogeneity of regression slopes assumption for ANCOVA was examined by testing the interaction between group and baseline scores. Estimated marginal mean differences and corresponding 95% confidence intervals (CIs) were reported to reflect the magnitude and precision of group differences. All statistical tests were two-tailed with a significance level set at *p* < 0.05. Effect sizes (partial eta squared, η^2^p) were reported for ANCOVA analyses.

### 3.2. Results

#### 3.2.1. Feasibility of the Pilot Study

Of the 52 individuals who expressed interest in the study, 45 met the eligibility criteria and provided informed consent. Following randomization, 15 participants in the CST group (68.18%) and 15 participants in the control group (65.22%) completed both pre- and post-intervention assessments and were included in the final analyses. Attrition after randomization was primarily related to child illness, hospitalization, and family relocation. All participants attended at least half of the intervention sessions. No serious adverse events or major implementation difficulties were reported during the intervention period. Intervention fidelity was supported through standardized operational procedures and formal fidelity assessment using a self-designed fidelity checklist and scoring system. Inter-rater reliability reached 85%.

#### 3.2.2. Differences Among Groups of Children

Baseline comparisons indicated no significant differences in the sex and age of children with autism between the CST and control groups, confirming group equivalence. In addition, no significant between-group differences were observed in baseline outcome measures (as shown in Table 5).

ANCOVA analyses, controlling baseline scores, revealed no significant group effects on the autism symptoms or any of the secondary outcomes at post-intervention (all *p* > 0.05). Effect sizes were small to moderate (η^2^p ranging from 0.000 to 0.067). For the autism symptoms, the ANCOVA revealed no statistically significant group difference at post-intervention after controlling for baseline scores, *F*(1, 26) = 1.88, *p* = 0.182, η^2^p = 0.067. However, the estimated marginal mean difference indicated a tendency toward lower post-intervention scores in the CST group compared to the control group (estimated marginal mean difference = −4.84, 95% CI [−9.33, −0.36]), which implies a potential beneficial effect on the autism symptom. Similarly, for the sensory/cognitive awareness subscale, the ANCOVA revealed no statistically significant group difference at post-intervention after controlling for baseline scores, *F*(1, 26) = 0.51, *p* = 0.481, η^2^p = 0.019. The estimated marginal mean difference suggested lower scores in the CST group compared to the control group (estimated marginal mean difference = −1.80, 95% CI [−3.52, −0.07]), suggesting a potential benefit for the sensory/cognitive awareness.

No statistically significant group differences were observed for sociability, *F*(1, 26) = 0.01, *p* = 0.931, η^2^p = 0.000, or health/physiology/behavior, *F*(1, 26) = 0.47, *p* = 0.499, η^2^p = 0.018. The estimated marginal mean differences for these outcomes were small and imprecise, with confidence intervals spanning zero (sociability: −0.98, 95% CI [−3.33, 1.37]; health/physiology/behavior: −0.84, 95% CI [−3.39, 1.72]). For the speech/language/communication, the ANCOVA revealed a significant interaction between group and baseline scores, *F*(1, 26) = 6.33, *p* = 0.018, η^2^p = 0.196, indicating that the effect of group varied as a function of baseline levels. Given this interaction, the main effect of group was not interpreted further. To further probe this interaction, a moderation analysis was conducted using PROCESS 3.4 (Model 1). The results confirmed a significant interaction between group and pre-intervention scores of speech/language/communication (A1), *b* = 0.29, *SE* = 0.12, *p* = 0.018. Conditional effects analysis showed that the group difference was not significant at low levels of A1 (*p* = 0.238), but became significant at moderate (*b* = 1.73, *p* = 0.003) and high levels (*b* = 2.97, *p* < 0.001) of A1. This pattern suggests that the intervention effect was stronger among participants with higher baseline scores.

Supplementary within-group analyses indicated that children in the CST group showed significant pre–post changes in speech/language/communication (*t* = 4.01, *p* < 0.001), sensory/cognitive awareness (*t* = 3.34, *p* < 0.01), and overall autism symptoms (*t* = 4.06, *p* < 0.001), where lower scores indicated better ability. No significant changes were observed in sociability or health/physiology/behavior. In contrast, the control group did not show significant pre–post changes in any outcome measures.

#### 3.2.3. Differences Among Groups of Caregivers

Baseline comparisons indicated no significant differences on the sex, age and mental health (GHQ), self-efficacy (CSQ), or parental stress (PSI-SF) of caregivers between the CST and control groups, confirming group equivalence (as shown in Table 6). However, a significant baseline difference was observed for caregiver knowledge and skills (WHO-CKST) (*t* = 2.28, *p* = 0.031).

ANCOVA analyses, controlling for baseline scores, revealed no significant group effects on caregiver mental health, self-efficacy, or parenting stress at post-intervention (all *p* > 0.05). Effect sizes were small (η^2^p ranging from 0.002 to 0.067). For caregiver mental health (GHQ), the ANCOVA revealed no statistically significant group difference at post-intervention after controlling for baseline scores, *F*(1, 26) = 1.86, *p* = 0.184, η^2^p = 0.067. The estimated marginal mean difference suggested lower scores in the CST group compared to the control group (estimated marginal mean difference = −0.36, 95% CI [−2.86, 2.15]); however, the wide confidence interval spanning zero suggests considerable uncertainty around the estimate. Similarly, no significant group differences were found for caregiver self-efficacy (CSQ), *F*(1, 26) = 0.31, *p* = 0.582, η^2^p = 0.012, or parenting stress (PSI-SF), *F*(1, 26) = 0.06, *p* = 0.813, η^2^p = 0.002. The estimated marginal mean differences for these outcomes were associated with wide confidence intervals spanning zero, indicating substantial uncertainty in the effect estimates (self-efficacy: 3.26, 95% CI [−0.13, 6.64]; parenting stress: −8.39, 95% CI [−13.91, 2.90]). For caregiver knowledge and skills (WHO-CKST), no significant group effect was observed after controlling for baseline scores, *F*(1, 26) = 0.32, *p* = 0.580, η^2^p = 0.012. Although adjusted mean comparisons suggested higher post-intervention scores in the CST group (mean difference = 15.25, 95% CI [9.60, 20.91]), this difference was not statistically significant.

Supplementary within-group analyses indicated that caregivers in the CST group showed significant changes in knowledge and skills (*t* = −5.75, *p* < 0.001) and reductions in parental stress (*t* = 2.35, *p* < 0.05), whereas no significant changes were observed in mental health or self-efficacy. In contrast, caregivers in the control group showed a decline in knowledge and skills over time (*t* = 2.34, *p* < 0.05).

## 4. Discussion

This study systematically examined the feasibility and modification of the WHO Caregiver Skills Training (CST) program within the Chinese cultural context. Guided by the ecological validity model (EVM), the training was culturally adapted to enhance its acceptability and applicability in the local setting. A pre- and post-test controlled trial was subsequently conducted to explore the feasibility of implementation and changes in caregiver- and child-related outcomes. Given the small pilot sample and the absence of statistically significant between-group effects for most outcomes after baseline adjustment, the findings should be interpreted cautiously as exploratory evidence rather than definitive evidence of intervention effectiveness.

### 4.1. Cultural Adaptation and Modification of CST in Enhancing Clarity, Comprehension, and Caregiver Acceptance

The multi-stakeholder consultation process was a key strength of Phase 1. By integrating perspectives from caregivers, professionals, and community representatives, the modification process was informed by clinical standards, implementation feasibility, and local sociocultural realities. Consistent with previous work emphasizing stakeholder involvement in intervention adaptation (Hoekstra et al., 2018), these findings suggest that locally relevant materials, examples, and delivery approaches may support caregiver engagement and the application of learned strategies within everyday routines.

Several adaptations were implemented based on qualitative feedback obtained during Phase 1. For example, technical terminology used in the original CST materials was simplified into more colloquial and culturally familiar language, and locally relevant examples and case illustrations were incorporated to improve practical understanding and contextual relevance. In addition, the delivery format and training schedules were adapted to better accommodate caregivers’ childcare responsibilities and daily routines through a flexible combination of online and offline sessions. This finding is consistent with WHO adaptation guidance and reports from other CST implementation contexts, which suggest that childcare support can be important for reducing attendance barriers and supporting caregiver participation. The use of a WeChat-based communication group may be understood as a context-specific implementation support that leveraged an existing and widely used social media tool to facilitate ongoing contact, rather than as a newly developed digital intervention platform. These adaptations may have contributed to the relatively high recruitment, retention, and caregiver engagement observed during the pilot implementation phase; however, additional contextual factors, such as family resources, service accessibility, caregiver availability, and broader socioeconomic conditions, may also have influenced participation and retention outcomes (Arakelyan et al., 2019; Hackworth et al., 2018; Li et al., 2023).

Similar findings have been reported in other culturally adapted caregiver-mediated interventions. Studies conducted in non-English-speaking countries often require language-level adjustments to improve understanding (Al Maskari et al., 2018; Buzhardt et al., 2016). Similarly, Albin et al. (2022) highlighted that effective cultural adaptation of parent-mediated interventions should address language use, caregiver roles, intervention delivery methods, and broader social environments to improve practical applicability. Parallel experiences from Ethiopia further demonstrated that community engagement and local stakeholder involvement contributed to the acceptability and sustainability of CST implementation and informed revisions for broader field testing (Tekola et al., 2020).

These findings highlight the potential value of culturally responsive adaptation in supporting caregiver participation and implementation feasibility within diverse sociocultural settings. Importantly, the qualitative adaptation findings provide a framework for interpreting the pilot outcomes rather than merely describing the modifications made to CST materials. Language simplification and the use of more familiar expressions may have reduced comprehension barriers and supported caregivers’ engagement with the training content. Locally relevant examples, demonstrations, and interaction scenarios may have helped caregivers connect CST strategies with everyday family routines, which may be particularly relevant to the descriptive changes observed in communication-related child outcomes. Flexible delivery arrangements and schedule adjustments may also have reduced participation barriers related to childcare responsibilities and daily demands, thereby contributing to recruitment and retention during the pilot phase. However, these proposed links were not directly tested in the present study and should be interpreted as exploratory explanations for future mixed-methods implementation research.

The qualitative findings further distinguish adaptations that aligned with existing WHO guidance from locally identified considerations that may add value for implementation in mainland China. While changes such as localizing names, terminology, examples, and culturally specific roles were consistent with WHO-recommended adaptations, stakeholders also highlighted additional context-specific issues, including intergenerational caregiving, family privacy during home visits, family goal alignment, time burden, and the perceived need for ongoing communication and peer exchange. These findings suggest that future adaptations of WHO CST should consider not only material-level cultural relevance but also family structure, service context, and sustained caregiver engagement, while carefully evaluating delivery-related modifications to preserve fidelity to core CST components.

### 4.2. Feasibility of Modified CST

Previous WHO CST implementation studies conducted across diverse cultural and resource settings have generally reported high recruitment and retention rates, although attrition patterns have varied by context. For example, urban implementation sites such as Hong Kong reported relatively high dropout rates associated with logistical barriers (Wong et al., 2022), whereas studies conducted in Ethiopia and India achieved near-complete retention through community-based delivery approaches and culturally responsive adaptations (Tekola et al., 2020; Sengupta et al., 2023). Similar implementation strategies have also been reported in Kenya, although retention outcomes remain pending (Washington-Nortey et al., 2024). Across studies, common reasons for attrition included structural and family-related challenges such as child illness and relocation.

In the present study, the culturally adapted and modified CST in mainland China also demonstrated relatively high recruitment and retention throughout the pilot phase. These feasibility patterns may be partly understood in relation to the Phase 1 adaptation findings, as flexible delivery arrangements and schedule adjustments were designed to accommodate caregivers’ childcare responsibilities and daily routines, thereby making the adapted intervention more acceptable and manageable for participating families within the local context. Nevertheless, participation outcomes were likely influenced by multiple contextual factors, including family circumstances, caregiver availability, service accessibility, and broader socioeconomic conditions. In addition, the use of modified operational procedures and fidelity checklist may have contributed to implementation consistency and participant engagement throughout the intervention process. Collectively, these findings suggest that culturally responsive adaptations, flexible implementation arrangements, and ongoing facilitator support may be important factors supporting the feasibility and sustainability of WHO CST implementation across diverse cultural and resource settings. However, the scalability implications of these feasibility findings should be interpreted cautiously. The pilot implementation relied on a relatively supported structure, including supervision by experienced Master Trainers, facilitator support, standardized procedures, and fidelity assessment. These supports may have contributed to implementation consistency but may not be readily available in routine service settings with limited workforce capacity or institutional resources. Moreover, implementation cost, facilitator workload, supervision requirements, service-system capacity, and long-term sustainability were not systematically assessed. Therefore, the present findings should be viewed as preliminary feasibility evidence under supported pilot conditions, while highlighting the need for further evaluation of workforce capacity, resource requirements, and sustainability before broader implementation.

### 4.3. Child Outcome Patterns

With respect to child outcomes, baseline-adjusted ANCOVA analyses did not show statistically significant between-group effects for most outcomes. Descriptive within-group changes were observed in speech/language/communication, sensory/cognitive awareness, and overall autism symptoms in the CST group; however, these findings should be interpreted as exploratory and hypothesis-generating rather than evidence of CST-specific intervention effects. The relatively modest between-group differences may also reflect the influence of the control condition, as children in the control group continued to receive regular curriculum-based intervention during the study period, which may have contributed to stability or gradual changes in some developmental domains. Notably, the speech/language/communication domain showed the most salient exploratory signal among the child outcomes, suggesting that baseline functioning in this domain may be an important source of variability in children’s response to CST. This finding should not be interpreted as confirmatory evidence of efficacy, but it indicates that communication-related outcomes and baseline child characteristics warrant closer examination in future adequately powered CST trials.

Previous CST studies have also reported variable effects across outcome domains, with relatively stronger signals often observed in communication-related outcomes than in broader social functioning. For example, a study conducted in Taiwan found that CST reduced autism symptoms but showed limited impact on social behaviors (Seng et al., 2022), while a randomized controlled trial in Italy demonstrated gains in non-verbal communication yet highlighted the need for stronger effects on social interaction (Salomone et al., 2022b). In the present study, the descriptive pattern and exploratory baseline-dependent finding may help inform future research by suggesting that communication-related outcomes may be particularly relevant for evaluating CST, although the current data are insufficient to conclude that CST produced superior child outcomes compared with the control condition.

One possible explanation is that communication and interaction strategies are directly targeted through caregiver-mediated practice and may be more readily embedded in daily family routines. The culturally adapted communication strategies and locally relevant interaction examples developed during Phase 1 may have helped caregivers understand and apply these practices more naturally in daily interactions with their children. This may partly explain why communication-related outcomes appeared more responsive than broader sociability outcomes in the present pilot study. However, this interpretation remains tentative, as mechanisms of change were not directly tested.

Broader sociability-related outcomes may require more intensive, specialized, or longer-term intervention support. Unlike caregiver–child communication routines, sociability often involves more complex peer interaction skills, generalized social adaptation, and opportunities for social participation across settings, which may be less likely to change during a relatively brief pilot intervention (Settanni et al., 2024). Cultural and contextual factors may also shape these patterns. In the Chinese context, families may place relatively greater emphasis on academic achievement and cognitive development, which could influence caregivers’ priorities toward parent–child communication and interaction rather than broader peer-related social participation (Hou et al., 2023; Xu et al., 2025). Therefore, the potential contribution of cultural and contextual factors to sociability-related outcomes warrants further investigation in future research.

### 4.4. Caregiver-Related Outcome Patterns

For caregiver-related outcomes, baseline-adjusted between-group differences were largely nonsignificant. Although supplementary within-group analyses indicated changes in caregiver knowledge and skills and parenting stress in the CST group, these findings should be interpreted descriptively and should not be taken as evidence of CST-specific effects. The relatively modest between-group differences may partly reflect the nature of the control condition. Caregivers in the control group received a 10-week educational program and continued to receive routine institution-based services, including daily feedback about their children from professionals during the study period. Such exposure may have contributed to changes or stability in some caregiver-related outcomes, thereby reducing observable group differences.

At the same time, the CST and control conditions were not fully equivalent in content, delivery format, and caregiver engagement demands. Whereas the control condition was primarily didactic and information-based, the CST group involved face-to-face group sessions combined with home visits or online meetings that emphasized active skill practice, modeling, individualized feedback, and application of strategies within daily routines. These differences may have influenced caregiver expectations, engagement, and opportunities to practice intervention strategies, introducing potential dosage- and engagement-related confounds. Therefore, the observed trends should be interpreted cautiously and cannot be attributed solely to CST-specific effects. Future trials should consider using more closely matched active control conditions or systematically measuring intervention dosage, caregiver engagement, and practice quality to better isolate CST-specific mechanisms.

Previous CST studies conducted in other cultural contexts have also reported variable caregiver-related outcomes. For example, culturally adapted CST in India was associated with changes in caregiver competencies (Sengupta et al., 2023), while research in Hong Kong reported reductions in parenting stress but limited changes in self-efficacy and psychological well-being (Lau et al., 2022). In the present study, the descriptive changes in caregiver knowledge, skills, and parenting stress may be useful for generating hypotheses and refining outcome selection for future trials, but they are insufficient to establish intervention efficacy.

Several factors may help explain the observed caregiver-related patterns. The flexible delivery arrangements and culturally adapted communication approaches identified during the qualitative adaptation of phase 1 may have supported caregiver engagement and perceived accessibility of the intervention. In addition, the apparent reduction in parenting stress in the CST group may partly reflect peer support and professional guidance during group sessions (Dunst et al., 2014), which could have strengthened caregivers’ perceived support and coping resources. However, the limited changes in self-efficacy suggest that knowledge acquisition alone may not be sufficient to foster confidence in caregiving abilities. As Lorig et al. (2001) argued, self-efficacy is often strengthened through repeated successful experiences, sustained positive reinforcement, and opportunities to apply newly acquired skills in daily life. Although caregivers participating in CST may gradually develop greater proficiency in using intervention strategies, confidence in caregiving abilities may require a longer period of consistent practice and support than was provided in the present short-term pilot intervention. In addition, many caregivers continued to face substantial structural and emotional pressures during the intervention period, including ongoing caregiving demands and the persistent economic burden associated with raising a child with autism. These broader contextual stressors may have limited the extent to which changes in knowledge and skills translated into measurable gains in self-efficacy and psychological well-being.

An additional finding was the decline in caregiver knowledge and skills observed in the control group over time. This pattern should be interpreted cautiously given the small sample size and limited statistical power of the present study. One possible explanation is that caregivers in the control group did not receive ongoing structured practice, feedback, or reinforcement during the intervention period, which may have influenced their confidence or consistency in applying caregiving strategies over time. In addition, changes in caregivers’ self-perception and response calibration may also have contributed to this pattern. However, these interpretations remain tentative and require further investigation in larger-scale studies.

This study provides preliminary evidence regarding the cultural adaptation and feasibility of implementing the WHO CST within the Chinese context, while also contributing to the broader cross-cultural literature on CST implementation. The findings are consistent with previous research suggesting that caregiver-mediated interventions often require adaptation to local language use, family structures, educational expectations, and service contexts to improve cultural relevance and acceptability. Although WHO CST was originally developed as a globally scalable intervention framework, culturally responsive modifications may still be necessary to facilitate implementation across diverse sociocultural settings. With respect to intervention outcomes, most baseline-adjusted between-group differences were not statistically significant. Therefore, the observed changes in child speech/language/communication, sensory/cognitive awareness, overall autism symptoms, caregiver knowledge and skills, and parenting stress should be interpreted as exploratory and hypothesis-generating rather than evidence of intervention efficacy. Given the pilot nature of the study, small sample size, reliance on caregiver-reported outcomes, and other methodological limitations, these findings should be interpreted cautiously. Future large-scale randomized controlled trials with longer follow-up periods, blinded and objective outcome assessments, and more robust statistical power are needed to evaluate the effectiveness of culturally adapted CST. Future research should also examine strategies to strengthen caregiver self-efficacy, identify subgroups that may benefit most, and develop more individualized intervention approaches across different cultural and resource settings.

### 4.5. Limitations and Future Recommendations

This study has several methodological limitations that should be considered when interpreting the findings. A key limitation of this pilot study is the reliance on caregiver-reported measures, such as the ATEC, for assessing both autism diagnosis and symptom changes, without inclusion of gold-standard instruments such as the ADOS or ADI-R. In addition, caregiver-related outcomes were also primarily based on self-report measures. Because caregivers were not blinded to group assignment, their reports may have been influenced by expectancy effects, social desirability, or personal investment in the intervention, potentially leading to biased or overestimated estimates of change. These factors may have limited the confidence in diagnostic confirmation and reduced the objectivity of outcome evaluation. Although the WHO CST is designed to be applicable without requiring a formal diagnosis, and caregiver-reported measures were considered appropriate for capturing preliminary changes in child behavior, communication, and social engagement during this pilot phase, these methodological factors may nevertheless have reduced confidence in diagnostic confirmation and increased the risk of biased effect estimation. Therefore, the present findings should be interpreted as preliminary and exploratory rather than definitive evidence of intervention efficacy. Future large-scale studies should incorporate standardized, independent, and blinded clinician-administered assessments, direct behavioral observations, and more objective behavioral outcome measures alongside caregiver reports, as these approaches are essential for reducing expectancy and reporting bias and strengthening the validity of intervention effect estimates.

Second, the relatively small sample size, post-randomization attrition, and complete-case analysis approach may have limited the statistical power and internal validity of the study. Only participants who completed both pre- and post-intervention assessments (*n* = 15 per group) were included in the final analyses, and attrition was primarily related to external factors such as child illness, hospitalization, and family relocation. Although ANCOVA was used to adjust for baseline scores and provide baseline-adjusted between-group comparisons, the small pilot sample and variability in baseline outcome measures may have reduced the stability and precision of the adjusted estimates. Therefore, the ANCOVA findings should be interpreted cautiously and considered exploratory rather than confirmatory evidence of intervention effects. The absence of formal missing-data analyses or imputation procedures may have increased the risk of unstable effect estimates and reduced the ability to detect significant between-group differences. In addition, the study did not conduct formal subgroup analyses examining variability across child age, symptom severity, caregiver characteristics, or family contextual factors, as such analyses would likely have been underpowered within the current pilot sample. Future studies with larger and more diverse samples are needed to better evaluate heterogeneity in intervention responses and strengthen both internal and external validity.

These limitations collectively constrain the internal validity of the study and reduce confidence that the observed changes can be attributed specifically to the CST intervention. Therefore, the findings should be interpreted as preliminary feasibility evidence and exploratory outcome signals rather than causal evidence of intervention efficacy.

Third, several implementation- and scalability-related limitations should also be acknowledged. Although intervention fidelity was supported through standardized operational procedures, a self-designed fidelity checklist, and inter-rater reliability assessment, the fidelity evaluation approach remained preliminary and was not externally validated. The modified delivery format also differed from the standard WHO CST model of nine group sessions and three individualized visits used in most previous CST evaluations, which may affect intervention fidelity, dosage, comparability with prior studies, and scalability. Moreover, the pilot implementation relied on a relatively intensive support structure, including supervision by experienced Master Trainers, facilitator support, standardized procedures, and fidelity assessment. While these components may have helped maintain implementation consistency during the pilot phase, they may also limit the generalizability of the findings to routine service settings where training, supervision, workforce capacity, and institutional resources may be more constrained.

In addition, the feasibility indicators reported in the present study were limited primarily to recruitment, retention, attendance, attrition reasons, adverse events, and fidelity assessment. Other important implementation outcomes, including caregiver acceptability, satisfaction, perceived appropriateness, implementation burden, intervention cost, workforce requirements, service-system capacity, and long-term sustainability, were not systematically assessed. Therefore, the feasibility findings should be interpreted as preliminary and limited to early indicators of participation and implementation continuity rather than as evidence of readiness for large-scale implementation.

While the Ecological Validity Model (EVM; Bernal et al., 1995) provided useful guidance for content and contextual adaptation, this framework does not fully address implementation science constructs such as reach, adoption, implementation burden, maintenance, or sustainability. Future research could integrate implementation science frameworks, such as RE-AIM or CFIR, and incorporate standardized implementation outcome measures and mixed-methods evaluations to more comprehensively assess caregiver experience, facilitator workload, cost, service-system capacity, sustainability, and scalability across diverse service settings.

The control condition was not fully matched to the CST group in terms of intervention content, delivery format, individualized feedback, and caregiver practice opportunities. Although the control group received substantial educational contact, differences in active caregiver engagement and skill-based coaching may have introduced treatment dosage and engagement-related confounds.

Furthermore, although several cultural and contextual interpretations were discussed in relation to caregiver priorities and sociability-related outcomes, the present study did not directly evaluate caregivers’ cultural beliefs, parenting values, or educational expectations. Therefore, these interpretations should be considered exploratory rather than empirically confirmed. Future qualitative and mixed-methods studies are needed to more directly examine how cultural and contextual factors may influence caregiver engagement, intervention implementation, and developmental outcomes in culturally adapted and modified CST.

Finally, the present study did not include a comprehensive assessment of intervention acceptability and feasibility from the perspectives of caregivers and other stakeholders. Future studies should incorporate broader implementation evaluation methods, including structured acceptability and feasibility measures, caregiver feedback, qualitative interviews, and longitudinal follow-up assessments, to further optimize culturally adapted and modified CST implementation and evaluate long-term intervention outcomes.

## 5. Conclusions

The pilot study provides preliminary evidence for the feasibility of a culturally adapted and modified WHO Caregiver Skills Training (CST) program in mainland China. Descriptive changes were observed in caregiver knowledge and skills, parenting stress, and selected child outcomes, including speech/language/communication, sensory/cognitive awareness, and overall autism symptoms. However, most between-group differences were not statistically significant after baseline adjustment. Given the pilot nature, small sample size, and methodological limitations of the study, the findings should be interpreted cautiously as preliminary evidence of feasibility under supported pilot conditions and as exploratory, hypothesis-generating outcome findings, rather than as evidence of efficacy or readiness for large-scale implementation. Future large-scale randomized controlled trials with longer follow-up, objective outcome measures, and greater statistical power are needed to evaluate effectiveness, sustainability, and implementation potential.

## Figures and Tables

**Table 1:** Demographics of participants in cultural adaptation of the WHO CST.

No	Gender	Age	Experience with ASD (Year)	Occupation/Identity	Interview Format	Interview Location
G1-P1 ^a^	Female	38	1	postgraduate student majoring in psychology	Focus group	Online meeting
G1-P2	Female	35	10	Therapist	Focus group	Online meeting
G1-P3	Female	34	6	Mother of children with autism	Focus group	Online meeting
G1-P4	Female	27	3	Therapist	Focus group	Online meeting
G1-P5	Female	37	1	Postgraduate student majoring in psychology	Focus group	Online meeting
G1-P6	Female	34	7	Mother of children with autism	Focus group	Online meeting
G1-P7	Female	35	3	Therapist	Focus group	Online meeting
G2-D1 ^b^	Female	50	>20	Child psychiatrist	Stakeholder consultation meeting	Rehabilitation institution
G2-T1	Male	55	>20	Directors of the rehabilitation institution	Stakeholder consultation meeting	Rehabilitation institution
G2-T2	Female	44	11	Therapist	Stakeholder consultation meeting	Rehabilitation institution
G2-T3	Female	37	5	Therapist	Stakeholder consultation meeting	Rehabilitation institution
G3-T1 ^c^	Male	52	6	Directors of the rehabilitation institution	Stakeholder consultation meeting	Rehabilitation institution
G3-T2	Female	36	3	Interventionists	Stakeholder consultation meeting	Rehabilitation institution
P1 ^d^	Female	35	7	Mother of children with autism	One-to-one interview	Rehabilitation institution
P2	Female	39	5	Mother of children with autism	One-to-one interview	Online Video call
P3	Male	43	9	Father of children with autism	One-to-one interview	Rehabilitation institution
P4	Male	32	4	Father of children with autism	One-to-one interview	Rehabilitation institution
P5	Female	34	6	Mother of children with autism	One-to-one interview	Rehabilitation institution
P6	Female	57	5	Grandma of children with autism	One-to-one interview	Rehabilitation institution
P7	Female	42	5	Mother of children with autism	One-to-one interview	Video call

^a^ indicates that G1 refers to the focus group. ^b^ indicates that G2 refers to the first stakeholder consultation meeting, which involved four participants. ^c^ indicates that G3 refers to the second stakeholder consultation meeting, which involved two participants. ^d^ indicates that P refers to the one-to-one in-depth interviews.

**Table 2:** Themes and sub-themes of cultural and contextual modifications, organized by EVM dimensions and FRAME-informed modification categories.

Dimensions	Themes and Sub-Themes	Primary Modification Focus Informed by FRAME
Persons	**1. The diversification of caregiver roles**	Caregiver participants and engagement support
	1.1 Participation of grandparents, family, and father 1.2 Caregiver differences	
Content	**2. Regional resource integration** 2.1 Regional resource requirements	Contextual content modification; linkage to local services
	**3. Knowledge of developmental psychology** 3.1 The need for knowledge of children’s development	Content clarification or addition
	**4. Content operation difficulties** 4.1 Persisting in execution and behavioral response are difficult 4.2 Flexible operation is required.	Delivery process modification; practice and implementation support
	**5. Family privacy protection** 5.1 Home visit privacy 5.2 Principles and theories 5.3 In-depth understanding 5.4 Reasonable expectations 5.5 Sharing of positive cases.	Privacy safeguards in delivery; content clarification; caregiver expectation and engagement support
	**6. Caregiver consciousness motivation** 6.1 Breaking stereotypes 6.2 Consistency of family goals 6.3 Cooperation between home and school, 6.4 Acceptance of the current situation 6.5 Objective assessment 6.6 Long-termism belief 6.7 Respect for individual differences	Content clarification and caregiver engagement support related to beliefs, expectations, family goal alignment, and sustained participation
	**7. Localization of examples** 7.1 Localization of the story, the characters, the scenes, and the cases	Intervention material modification through substitution or revision of stories, characters, scenes, and examples to improve local relevance
	**8. Specification of Goals** 8.1 Set specific goals and record the progress of goal implementation	Goal-setting and progress-monitoring support to facilitate caregiver practice and application in daily routines
	**9. The effectiveness of strategies** 9.1 Some strategy failure 9.2 Grandparent strategy 9.3 Difficulty adaptation	Strategy tailoring and practice support to address caregiver-reported implementation barriers while preserving core CST strategies
	**10. Module arrangement** 10.1 Remove duplicate content and integrate the modules 10.2 Make the content concise and focused, and express it in a localized manner.	Structural and delivery-related modification involving content condensation, module integration, and localized simplification; requires attention to fidelity
Methods	**11. Recruitment methods**	Recruitment and reach strategies; modification to participant identification, recruitment channels, and eligibility-related implementation procedures
	11.1 Challenges in recruitment, target demographics for recruitment, recruitment channels, forms of recruitment, training themes, and the age range of children	
	**12. Course structure design** 12.1 Theoretical and practical ratio 12.2 Course structure arrangement and content preview 12.3 Two-way feedback assessment 12.4 Caregiver needs communication 12.5 Problem-solving solutions	Delivery process and session structure modification; adjustment of theory-practice balance, session organization, feedback mechanisms, and problem-solving supports
	**13. Continuous support** 13.1 Temporary caregiver arrangements 13.2 Training behavior reinforcement 13.3 Regular feedback and community building	Implementation support and caregiver engagement strategies; ongoing reinforcement, feedback, and community-building supports to sustain caregiver participation and practice
	**14. Training scale** 14.1 Group optimization	Delivery-level modification related to group size, group composition, and training scalability
	**15. Training material** 15.1 Operation manual 15.2 Knowledge checklist	Training material and support-tool modification; addition or revision of manuals and checklists to support caregiver comprehension and independent practice
	**16. Time allocation** 16.1 Training duration and frequency 16.2 One-on-one feedback and relationship building 16.3 Group discussions on similar issues, and demonstration exercises	Structural and delivery-process modification involving training duration, frequency, individualized feedback, group discussion, and demonstration opportunities; requires attention to fidelity and practice dosage
	**17. Training formats** 17.1 Caregiver mutual aid groups and peer empowerment 17.2 Technology application, online communities, and round-robin speaking mechanisms	Proposed caregiver engagement and delivery support involving peer exchange, mutual aid, continuous communication, and structured participation mechanisms
	**18. Training location** 18.1 Online and offline preference	Delivery setting modification; adjustment of online, offline, or hybrid delivery settings to improve feasibility and accessibility
	**19. Follow-up course**	Proposed post-intervention support to maintain caregiver practice and engagement
Concepts	**20. Cultural Taboos—Stigma of Illness** 20.1 Not mentioning diagnosis and disease	Stigma-sensitive terminology and concept modification to improve cultural acceptability and reduce barriers to caregiver engagement
	**21. Concept and Terminology Adjustment** 21.1 Family routines—family rituals—family series of activities 21.2 Challenging behavior—Problem behavior—Inappropriate behavior	Terminology and concept clarification to improve semantic accuracy, caregiver comprehension, and local acceptability
Language	**22. Localization of names**	Linguistic and cultural tailoring of intervention materials through adaptation of character names to improve familiarity and local relevance
	**23. Localization of intergenerational terms of address**	Linguistic and cultural tailoring of kinship and intergenerational terms to improve semantic accuracy, cultural appropriateness, and caregiver comprehension
	**24. Make the language used to teach concepts more colloquial**	Plain-language and colloquial adaptation of concept explanations to improve caregiver comprehension and acceptability
Metaphors	**25. Cultural symbol adaptability** 25.1 Priesthood, illustrations, and religious topics	Intervention material modification through substitution or removal of culturally incongruent religious symbols, illustrations, and culturally sensitive examples to improve local acceptability
Context	**26. Childcare** 26.1 Childcare during caregiver training	Barrier-reduction and caregiver engagement support through childcare arrangements to improve attendance, accessibility, and participation feasibility
	**27. Social, economic, educational and environmental conditions** 27.1 Family division of labor influence 27.2 Educational environment and educational philosophy 27.3 Difficulties in grandparents’ discipline 27.4 Unexpected events and weather factors 27.5 Caregivers’ willingness to learn 27.6 Time cost	Contextual implementation support addressing family roles, educational beliefs, socioeconomic constraints, environmental barriers, caregiver motivation, and time burden to improve feasibility and sustained engagement
Goals	**28. Personalized assessment and goal setting** 28.1 Target matching degree 28.2 Baseline assessment 28.3 Goal setting and detection 28.4 One-on-one feedback	Individualized assessment, goal-setting, and feedback support to improve alignment between caregiver training goals, child baseline abilities, and family needs
	**29. Psychological needs of caregivers** 29.1 Caregiver fatigue, distress, anxiety, and anger 29.2 Psychological counseling for caregivers and group counseling	Caregiver well-being and supplementary psychosocial support needs; potential addition of caregiver emotional support requiring consideration of intervention scope and fidelity
	**30. Caregiver behavioral goals** 30.1 Parental learning behavior goals, intervention behavior goals, and behavior monitoring and reinforcement	Caregiver behavior-change goal setting, monitoring, and reinforcement to support acquisition and sustained use of CST strategies in daily routines

Note. Themes and sub-themes were developed through thematic analysis and organized according to EVM dimensions. The primary modification focus column was informed by selected FRAME elements, including what was modified, the rationale for modification, and potential fidelity considerations. This table does not represent full FRAME coding across all domains.

**Table 3:** Intervention sessions after adaptation.

CST Sessions	Topic	Integrated Sessions	Topics
Session 1	Introduction to the program and	Session 1	Basic introduction to the course and how to arrange the environment to encourage children’s participation in activities.
	Getting Children Engaged		
Session 2	Keeping Children Engaged	Session 2	Help children to continuously participate in games and family activities.
Session 3	Helping Children to Share Engagement in Play and Home Routines		
Session 4	Understanding Communication	Session 3	Strategies for understanding and facilitating communication and teaching new skills step by step.
Session 5	Promoting Communication		
Session 6	Teaching New Skills in Small Steps and Levels of Help		
Session 7	Preventing Challenging Behavior–Helping Children Stay Engaged and Regulated	Session 4	Prevention, Management and Response to Challenging Behaviors.
Session 8	Teaching Alternatives to Challenging Behavior		
Session 9	Problem Solving and Self-Care	Session 5	Set the next goals, problem-solving strategies, and self-care for caregivers.

**Table 4:** Participant demographics in pre- and post-intervention controlled trials.

Variables	CST Group (*n* = 15)	Control Group (*n* = 15)	Group Comparison ^d^
**Children**			
**Age ^a^**	6.15 ± 1.59	6.08 ± 1.29	*t* (28) = 0.128, *p* = 0.899
**Sex (male:female)**	10:5	10:5	χ^2^ (1) = 0.00, *p* = 1.00
**Intervention duration ^a^ (hour/week)**	14.53 ± 6.99	16.47 ± 8.17	
**Types of education receiving ^b^**			
Kindergarten	5 (33.3%)	4 (26.7%)	
Primary school	1 (6.7%)	1 (6.7%)	
Special education institutions	9 (60.0%)	10 (66.7%)	
**Caregivers**			
**Age ^c^**	35 (31, 41)	36 (32, 42)	*t* (28) = 0.021, *p* = 0.984
**Sex (male:female)**	1:14	2:13	*p* = 1.00
**Roles of caregivers ^b^**			
Father	1 (6.7%)	2 (13.3%)	
Mother	12 (80.0%)	10 (66.7%)	
Grandparents	2 (13.3%)	2 (13.3%)	
Others	0	1 (6.7%)	
**Education level ^b^**			
High school and below	7 (46.7%)	3 (20%)	
Diploma	3 (20%)	7 (46.7%)	
Bachelor	3 (20%)	4 (26.7%)	
Master and above	1 (6.7%)	1 (6.7%)	
**Monthly household income ^b^** **(Yuan)**			
5000 and below	3 (20%)	4 (26.7%)	
5000 to 9999	4 (26.7%)	6 (40.0%)	
10,000 to 15,000	8 (53.3%)	4 (26.7%)	
15,000 and above	0	1 (6.7%)	

^a^ indicates that age and intervention duration of children are the mean and standard deviation. ^b^ indicates that the number of participants and the percentage of total participants involved. ^c^ indicates the age of caregivers is presented as the mean and range. ^d^ Group differences were tested using independent-samples *t*-tests for continuous variables and chi-square tests or Fisher’s exact tests for categorical variables, as appropriate.

**Table 5:** Pre- and post-intervention outcomes of children (*n* = 30).

Variables	CST Pre	CST Post	Control Pre	Control Post	Baseline	Estimated Marginal Mean Difference	ANCOVA	Effect Size
	(M ± SD)	(M ± SD)	(M ± SD)	(M ± SD)	*t* (*p*)	(95% CI)	*F* (*p*)	η^2^p
Autism symptom ^a^	53.67 ± 14.54	48.2 ± 17.09	57.6 ± 13.65	57.2 ± 14.94	−0.764 (0.451)	−4.841 * [−9.327, −0.355]	1.882 (0.182)	0.067
Speech/Language/Communication ^b^	9.87 ± 6.23	8.47 ± 5.38	10 ± 3.74	10.6 ± 4.58	−0.071 (0.994)	-	-	-
Sociability	17.73 ± 6.35	16.33 ± 6.09	16.87 ± 4.45	16.87 ± 4.45	0.433 (0.669)	−0.981 [−3.333, 1.370]	0.008 (0.931)	0.000
Sensory/Cognitive awareness	11 ± 5.26	8.73 ± 4.68	12.5 ± 4.24	12 ± 4.94	−0.917 (0.367)	−1.798 * [−3.522, −0.074]	0.511 (0.481)	0.019
Health/Physiology/Behavior	15.07 ± 5.35	14.67 ± 5.78	14.67 ± 5.78	18.07 ± 7.16	−1.214 (0.235)	−0.835 [−3.393, 1.723]	0.471 (0.499)	0.018

Notes: *: *p* < 0.05. ^a^ indicates that the total scores of Autism Treatment Evaluation (ATEC). ^b^ indicates that the homogeneity of regression slopes assumption was violated for this outcome; therefore, the ANCOVA main effect is not reported. A significant group × baseline interaction was observed.

**Table 6:** Pre- and post-intervention outcomes of caregivers (*n* = 30).

Variables	CST Pre	CST Post	Control Pre	Control Post	Baseline	Estimated Marginal Mean Difference ^a^	ANCOVA	Effect Size
	(M ± SD)	(M ± SD)	(M ± SD)	(M ± SD)	*t* (*p*)	(95% CI)	*F* (*p*)	η^2^p
GHQ	8.73 ± 4.83	7.60 ± 5.57	7.8 ± 5.38	7.2 ± 4.89	0.500 (0.621)	−0.356 * [−2.862, 2.150]	1.864 (0.184)	0.067
WHO-CKST	91.33 ± 6.82	100.60 ± 7.58	86 ± 5.96	81.47 ± 8.63	2.280 * (0.031)	15.252 *** [9.595, 20.909]	0.315 (0.580)	0.012
CSQ	50.73 ± 6.63	52.53 ± 7.59	46.6 ± 7.32	45.73 ± 6.91	1.622 (0.116)	3.259 [−0.125, 6.643]	0.310 (0.582)	0.012
PSI-SF	93.27 ± 18.51	87.53 ± 14.82	95.67 ± 17.67	97.73 ± 15.87	−0.363 (0.719)	−8.392 ** [−13.914, 2.896]	0.057 (0.813)	0.002

Notes: *: *p* < 0.05; **: *p* < 0.01; ***: *p* < 0.001. GHQ: The General Health Questionnaire; WHO-CKST: WHO caregiver knowledge and skills test; CSQ: Caregiver self-efficacy questionnaire; PSI-SF: Parenting stress index/short form. ^a^ indicates that the homogeneity of regression slopes assumption was violated for this outcome; therefore, the ANCOVA main effect is not reported. A significant group × baseline interaction was observed.

## Data Availability

The data that support the findings of this study are available from the corresponding authors upon reasonable request.
